# Establishment and application of an infection prevention and control system for patients undergoing continuous renal replacement therapy

**DOI:** 10.3389/fmed.2025.1506188

**Published:** 2025-07-07

**Authors:** Zhongjing Zhao, Huijie Zhao, Yang Yang, Yuncong Wang, Xia Zhao

**Affiliations:** Division of Healthcare-Associated Infection Management, Xuanwu Hospital, Capital Medical University, Beijing, China

**Keywords:** continuous renal replacement therapy, infection prevention and control, nursing quality, Delphi method, clinical management

## Abstract

**Background:**

Continuous renal replacement therapy (CRRT) is essential for critically ill patients but carries a high risk of hospital-acquired infections. Implementing an effective infection prevention and control system is critical for improving patient safety.

**Methods:**

This study utilized the Delphi method to develop a comprehensive infection prevention and control indicator system specifically designed for CRRT patients. A total of 126 patients treated at Xuanwu Hospital, Capital Medical University from January to December 2023 were included in the study. They were divided into a control group (*n* = 62), which received standard care, and an observation group (*n* = 64), which adopted the newly developed indicator system. Adverse event rates and nursing quality scores were compared between the two groups.

**Results:**

The final indicator system consisted of 3 primary, 9 secondary, and 27 tertiary indicators, with expert consensus reflected by a reliability coefficient of 0.795. The observation group demonstrated a significantly lower incidence of adverse events (3.12% vs. 16.12%, *P* < 0.05) and higher nursing quality scores across three dimensions (basic care, equipment management, and nursing safety) compared to the control group (*P* < 0.05).

**Conclusion:**

The infection prevention and control indicator system for CRRT patients exhibited strong reliability and scientific validity. Its implementation led to a significant reduction in infection rates and improvements in nursing quality, highlighting its potential for broader clinical adoption.

## 1 Introduction

Continuous renal replacement therapy (CRRT) is a pivotal intervention for managing acute kidney injury (AKI) in critically ill patients, particularly those who are hemodynamically unstable. CRRT offers superior fluid management, metabolic control, and the removal of large toxins and cytokines compared to intermittent dialysis (1, 2). It supports aggressive nutritional and inotropic strategies without worsening azotemia or fluid overload (3). Additionally, CRRT may enhance hemodynamics and gas exchange in septic shock patients, independent of fluid balance effects (4). Despite its benefits, the complexity and invasive nature of CRRT increase the risk of hospital-acquired infections, especially catheter-related bloodstream infections (CRBSIs), which can extend hospital stays, escalate morbidity, and inflate healthcare costs (5).

To mitigate these risks, evidence-based infection prevention protocols and strict adherence by nursing staff are essential (6). Specialized ICU nursing quality control programs have shown efficacy in improving CRRT outcomes by reducing unplanned events, CRBSIs, and costs, while enhancing patient satisfaction (7). However, the high incidence of central line-associated bloodstream infections (CLABSIs) in CRRT patients, particularly in critically ill children, remains a significant concern (8). Effective prevention strategies include rigorous hand hygiene, maximal barrier precautions, chlorhexidine skin antisepsis, optimal catheter site selection, and daily review of line necessity (9). Risk factors such as femoral vein catheterization, prolonged catheter indwelling time, compromised immune function, and high APACHE II scores contribute to increased infection risk, particularly when CRRT duration exceeds 4.5 days (8, 10). Despite existing measures, the incidence of hospital-acquired infections underscores the need for a comprehensive and standardized infection control system tailored to CRRT. Variability in nursing practices due to different levels of training and adherence to protocols exacerbates this issue, highlighting the necessity for standardized protocols and guidelines to ensure consistent best practices across healthcare providers.

This study aims to develop and evaluate a comprehensive infection prevention and control indicator system specifically designed for CRRT patients. Employing the Delphi method, this research gathered insights from experts in nephrology, critical care nursing, and infection control through a structured, multi-round survey process. This iterative feedback refined and prioritized indicators for the infection prevention system, ensuring its clinical relevance and applicability. The study design included a comparative analysis between a control group receiving standard care and an observation group implementing the new indicator system. By comparing the incidence of adverse events and evaluating nursing quality scores between these groups, the effectiveness of the indicator system in reducing infections and enhancing care quality was systematically analyzed. This approach not only validates the system but also provides actionable insights for improving infection control practices in CRRT settings, aiming to enhance patient safety and care quality in critical care environments.

## 2 Materials and methods

### 2.1 Study design

This study utilized a prospective, randomized controlled trial design to evaluate the effectiveness of a comprehensive infection prevention and control indicator system for patients undergoing CRRT. The trial was conducted at Xuanwu Hospital, Capital Medical University over a 1-year period, from January to December 2023. By randomly assigning eligible patients to either a control group receiving standard care or an observation group implementing the new indicator system, the study aimed to provide robust evidence on the impact of the intervention on infection rates and nursing quality.

### 2.2 Participants

Inclusion criteria: participants in the study were selected based on the following criteria: Age: patients aged 18 years and older. Diagnosis: individuals diagnosed with AKI or other critical conditions requiring CRRT. Patients must have been on CRRT for at least 3 days to ensure sufficient exposure to the treatment. Consciousness and communication: patients needed to be alert and able to communicate effectively to participate in the study. Exclusion criteria: patients were excluded from the study if they met any of the following conditions: Malignancies: individuals with a diagnosis of malignant tumors. Severe organ dysfunction: patients exhibiting severe dysfunction of other vital organs (e.g., liver failure and respiratory failure) that could complicate the management of CRRT. Immunocompromised status: patients with conditions that significantly impair the immune system, such as advanced HIV/AIDS or those on immunosuppressive therapy. History of shock: a history of septic or hypovolemic shock prior to CRRT initiation. Refusal to participate: patients who declined to provide informed consent for participation in the study.

### 2.3 Group allocation

Participants were randomly allocated to either the control group or the observation group using a computer-generated randomization sequence. Control group: patients assigned to this group received standard care practices, which included routine infection prevention measures and nursing management without the implementation of the newly developed infection prevention and control indicator system. Sample size: *n* = 62.

Observation group: patients in this group were provided with care according to the comprehensive infection prevention and control indicator system specifically designed for CRRT patients. This group received enhanced protocols aimed at reducing infection rates and improving nursing quality. Sample size: *n* = 64. The allocation resulted in a total of 126 participants, with a balanced distribution between the two groups.

### 2.4 Control group management

Patients in the control group received standard care practices, which included the following key components to manage infection risk during CRRT: Environmental management: maintenance of a clean and sterile environment in the CRRT unit to minimize exposure to pathogens. Regular cleaning and disinfection protocols were followed for all surfaces and equipment. Adherence to appropriate air quality standards, including regular ventilation checks and monitoring of room temperature and humidity levels. Nursing training: all nursing staff underwent comprehensive training sessions on standard infection prevention protocols. This training covered topics such as hand hygiene, proper use of personal protective equipment (PPE), and techniques for sterile catheter insertion and maintenance. Ongoing education was provided to ensure staff remained updated on best practices in infection control and management of CRRT patients. Risk stratification: patients were assessed for their individual risk factors for infection, including underlying health conditions, nutritional status, and previous history of infections. This risk stratification informed tailored care approaches within the standard protocol. Implementation of regular monitoring for early signs of infection, allowing for prompt identification and intervention if necessary. Standardized care protocols: utilization of standardized nursing protocols for the management of CRRT, including guidelines for catheter care, fluid management, and monitoring for complications. Regular audits and feedback sessions to ensure adherence to these protocols, with corrective actions taken as needed to maintain high standards of care.

### 2.5 Observation group management

In the observation group, the comprehensive infection prevention and control indicator system was implemented with the following key components: Indicator system implementation: the system included specific, measurable indicators across three levels: structural, process, and outcome indicators, aimed at enhancing infection prevention and patient safety during CRRT. Indicators covered areas such as catheter care, environmental hygiene, and staff compliance with infection control protocols, allowing for ongoing monitoring and evaluation. Training for nursing staff: a series of training workshops were conducted to educate nursing staff on the new protocols associated with the indicator system. These sessions included both theoretical and practical components, ensuring comprehensive understanding and application of the guidelines. Training materials included detailed manuals, visual aids, and scenario-based simulations that illustrated best practices in infection prevention specific to CRRT. Staff participated in hands-on training to practice new skills, such as enhanced catheter maintenance techniques and proper use of PPE, under the supervision of experienced trainers. Ongoing support and feedback: a mentorship program was established, pairing less experienced nurses with seasoned staff to provide guidance and support during the initial implementation phase. Regular feedback sessions were held to discuss challenges, successes, and areas for improvement in adhering to the new protocols, fostering a culture of continuous learning and quality enhancement. Monitoring and evaluation: the implementation of the indicator system included regular audits to assess compliance with the new protocols, ensuring that nursing staff maintained high standards of care throughout the study period. Performance metrics based on the established indicators were used to provide actionable insights, allowing for timely adjustments and interventions as needed.

### 2.6 Indicator system development

Delphi method process: the Delphi method was employed as a structured communication technique to achieve consensus among experts in the field of infection prevention and CRRT. This iterative process involved multiple rounds of questionnaires, allowing experts to review and refine their responses based on the feedback from previous rounds. Initially, a set of potential indicators was developed from a comprehensive literature review. Experts were then asked to rate the importance of each indicator on a Likert scale. After the first round, the results were analyzed, and indicators that received low scores or high variability were revised or eliminated. The process was repeated for two rounds, during which experts had the opportunity to reassess their opinions and consider the group’s feedback, leading to a refined list of indicators that achieved a higher level of agreement. Expert panel composition: the expert panel consisted of five members with diverse expertise relevant to CRRT and infection control. The composition included: one head nurse from the blood purification unit. One deputy chief physician specializing in nephrology. One specialized nurse with extensive experience in blood purification. One nursing researcher with a focus on quality improvement. One academic expert in infection control practices. Selection criteria: experts were selected based on their qualifications, including: a minimum of 5 years of clinical or research experience in CRRT or related fields. Academic credentials, such as a master’s degree or higher in nursing, medicine, or a related discipline. Demonstrated knowledge and expertise in infection prevention and control practices. Identification, refinement, and finalization of indicators: initially, potential indicators were identified through literature searches and existing guidelines on infection prevention in CRRT. A preliminary pool included structural, process, and outcome indicators relevant to patient safety and infection control. During the first Delphi round, experts evaluated the importance and feasibility of each indicator. Based on their feedback, a subset of indicators was refined to address overlapping concepts and enhance clarity. In the second round, experts further refined the indicators, focusing on practicality and clinical relevance. Indicators that achieved an importance score of at least 3.5 and had a low coefficient of variation (CV) were included in the final system. The finalized indicator system consisted of three primary indicators, nine secondary indicators, and 27 tertiary indicators, all aimed at enhancing infection prevention strategies for CRRT patients.

### 2.7 Data collection

Data collection methods for adverse events: adverse events related to CRRT, including catheter-related infections, accidental disconnections, and other complications, were systematically monitored and recorded over the course of the study. Nursing staff documented any adverse events in patient records, which were reviewed by the research team at the end of the intervention period. The collection process involved: daily monitoring of patients for signs of infection, such as fever, redness at the catheter site, or other clinical manifestations. Weekly audits of patient records to identify and confirm reported adverse events. Nursing quality assessments: nursing quality was assessed using a specifically designed questionnaire that encompassed three key dimensions: basic nursing care, equipment management, and nursing safety. The evaluation aimed to measure the quality of care provided to patients in both the control and observation groups. Tools and questionnaires used: Continuous Blood Purification Nursing Quality Evaluation Scale: this scale consisted of 30 items scored on a Likert-type scale ranging from 0 to 4, where higher scores indicated better nursing quality. The scale was designed to evaluate: Basic nursing care: aspects such as patient assessment, hygiene practices, and educational support provided to patients and families. Equipment management: evaluation of equipment maintenance, proper usage of devices, and adherence to sterilization protocols. Nursing safety: assessment of protocols for monitoring patients, managing emergencies, and preventing adverse events. The questionnaire underwent a validation process to ensure reliability, with a Cronbach’s alpha coefficient calculated at 0.868, indicating strong internal consistency.

Data analysis: collected data on adverse events and nursing quality scores were compiled and analyzed using statistical software (SPSS 20.0). Descriptive statistics were employed to summarize the data, while comparative analyses (e.g., Chi-square tests for categorical variables) were performed to assess differences between the control and observation groups.

### 2.8 Statistical analysis

Data analysis was conducted using SPSS version 20.0 and Excel 2010. SPSS was utilized for advanced statistical testing, while Excel was used for data organization and preliminary calculations. To compare groups, the following statistical tests were used: Chi-square tests: employed to analyze categorical data, such as the incidence of adverse events between the control and observation groups. Independent *t*-tests: used to compare continuous variables, such as nursing quality scores, between the two groups. Descriptive statistics: means and standard deviations were calculated for continuous variables, while frequencies and percentages were determined for categorical data. A significance level of α = 0.05 was established for all statistical tests. *P* values less than 0.05 were considered indicative of statistically significant differences between the control and observation groups.

### 2.9 Ethical considerations

The study received ethical approval from the hospital’s ethics committee (Approval No. 20230152). This ensured that the research adhered to ethical standards concerning patient safety and welfare. Informed consent was obtained from all participants prior to their inclusion in the study.

## 3 Results

### 3.1 Patient recruitment and selection process

**FIGURE 1:**
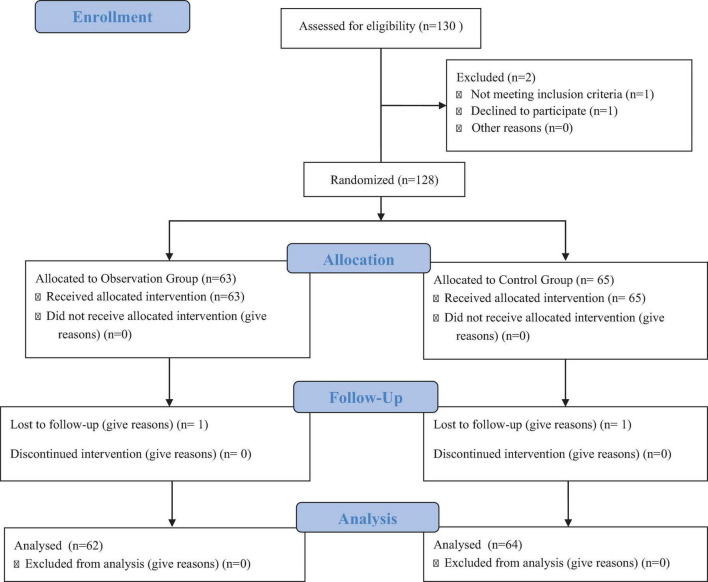
Flowchart of patient enrollment, allocation, follow-up, and analysis.

A total of 130 patients undergoing CRRT from January to December 2023 were assessed for eligibility in the study. The inclusion criteria required patients to have received CRRT treatment, undergone blood purification for more than 3 days, had an expected survival time exceeding 1 year, demonstrated clear consciousness and the ability to communicate effectively, cooperated with nursing protocols, and provided informed consent for participation. Patients were excluded if they had malignancies, severe organ dysfunction, immune system disorders, coagulation dysfunctions, or a history of shock. Out of the 130 patients assessed, 2 were excluded—1 for not meeting the inclusion criteria and 1 who declined to participate. This left 128 patients who were randomized into two groups: 63 were allocated to the Observation Group and 65 to the Control Group. All patients in both groups received the allocated intervention. During the follow-up, one patient from each group was lost to follow-up. No patients discontinued the intervention. In the final analysis, 62 patients from the Observation Group and 64 from the Control Group were included (Figure 1).

### 3.2 Demographics and clinical characteristics of participants

The control group consisted of 62 patients (35 males and 27 females), with an average age of 48.45 ± 5.14 years and a mean duration of CRRT of 4.13 ± 1.16 months. Disease classification revealed 28 patients with sepsis, 18 with multiple organ failure, and 16 with other conditions. The observation group included 64 patients (36 males and 28 females), with a slightly higher average age of 48.90 ± 5.08 years and a similar mean CRRT duration of 4.09 ± 1.21 months. This group also comprised 30 patients with sepsis, 19 with multiple organ failure, and 15 with other diagnoses. Statistical analysis indicated no significant differences in sex, age, duration of CRRT, or disease classification between the two groups (*P* > 0.05) (Table 1).

**TABLE 1:** General information of research subject (x̄ ± s).

Group	Control group	Observation group	*t*	*P*
Male/female	35/27	36/28	0.236	0.687
Age	48.45 ± 5.14	48.90 ± 5.08	0.156	0.831
Duration (month)	4.13 ± 1.16	4.09 ± 1.21	0.255	0.605
**Disease**				
Sepsis	28	30	0.026	0.895
Multiple organ failure	18	19	0.037	0.776
Others	16	15	0.035	0.778

### 3.3 Results of the Delphi method consultation

The expert consultation process revealed a high level of authority and engagement among the panelists. The response rates for the first and second rounds of the consultation were 100% and 98%, respectively, reflecting high participant engagement (positive coefficients of 1.00 and 0.98). Kendall’s *W*: this value measures the level of agreement among experts during the Delphi rounds. The closer the value is to 1, the stronger the agreement among experts. A value of 0.246–0.358, as reported in our study, indicates a moderate level of consensus, which is typical in Delphi studies where expert opinions may vary but converge over multiple rounds. The significant statistical result (*P* < 0.05) suggests that the expert panel reached a level of agreement beyond chance. Chi-square tests: the Chi-square test was used to analyze the distribution of categorical variables, such as the incidence of adverse events between the control and observation groups. A significant result (*P* < 0.05) indicates that there is a statistically significant difference in the frequencies of adverse events between the two groups, supporting the effectiveness of the intervention (Table 2).

**TABLE 2:** Kendall’s concordance coefficient for the coordination of expert consultation opinions in two rounds.

	First round inquiry			Second round inquiry	
*W* value	χ^2^	*P* value	*W* value	χ^2^	*P* value
0.285	29.763	<0.001	0.358	37.373	<0.001
0.276	41.635	<0.001	0.246	40.954	<0.001

### 3.4 Results of the Delphi method consultation

Based on the results of the expert consultations, the research team established a comprehensive set of infection prevention and control indicators tailored for patients undergoing CRRT. This framework consists of 3 primary indicators, 9 secondary indicators, and 27 tertiary indicators. The importance scores for these indicators, along with their coefficients of variation, are presented in Table 3. The primary indicators, which include structural, process, and outcome indices, received high importance scores, reflecting their critical role in enhancing infection control measures. Notably, the institutional norms and nursing operator indicators scored 4.85 ± 0.13 and 4.87 ± 0.15, respectively, indicating strong expert consensus on their significance. The secondary and tertiary indicators also demonstrated substantial importance, with scores ranging from 4.54 to 4.88 across various metrics. The coefficients of variation were relatively low, indicating consistency in the experts’ evaluations. This structured indicator system aims to provide a robust framework for monitoring and improving infection control practices in CRRT patients, thereby potentially enhancing patient outcomes (Table 3).

**TABLE 3:** Evaluation indicators for infection prevention and control in patients undergoing continuous blood purification (x̄ ± s).

Primary indicator	Secondary indicator	Importance score	Tertiary indicator	Importance score	Coefficient of variation
Structural index	Institutional norm	4.55 ± 0.26	Nursing practice	4.54 ± 0.23	0.050
			Complication prevention norms	4.54 ± 0.23	0.050
			Adverse event prevention guidelines	4.60 ± 0.21	0.049
	Nursing operator	4.66 ± 0.23	Working years ≥ 5 years	4.68 ± 0.21	0.043
	Equipment environment	4.58 ± 0.24	Obtained the qualification	4.69 ± 0.20	0.042
			Environmental health monitoring qualified	4.56 ± 0.23	0.050
			The device is running properly	4.60 ± 0.24	0.046
Process index	Patient evaluation	4.85 ± 0.13	Evaluation of contraindications and indications	4.85 ± 0.11	0.021
			Evaluation of coagulation function	4.83 ± 0.12	0.023
			Capacity and internal environment status assessment	4.81 ± 0.13	0.025
	Preparation for treatment	4.87 ± 0.15	Filter, pipe type selection	4.76 ± 0.14	0.029
			Equipment status check	4.86 ± 0.10	0.021
			Choice of treatment	4.80 ± 0.12	0.025
			Install filter matching pipes	4.82 ± 0.11	0.023
			Connecting blood vessel, solution bag and anticoagulant	4.84 ± 0.11	0.023
			Heparin saline preflush piping and blood filter	4.87 ± 0.08	0.016
			Sterilization of deep vein puncture tubes	4.79 ± 0.13	0.027
			Catheter fixation and functional evaluation	4.81 ± 0.12	0.025
	Extract blood for machine	4.88 ± 0.16	Setting treatment parameters	4.77 ± 0.13	0.027
			Sign, volume, hemodynamic monitoring	4.88 ± 0.07	0.014
			Machine alarm handling	4.82 ± 0.12	0.024
Outcome index	Anticoagulation complication	4.85 ± 0.14	Incidence of bleeding	4.86 ± 0.09	0.019
	Catheter complication		Incidence of coagulation	4.88 ± 0.07	0.013
			Incidence of infection	4.87 ± 0.07	0.014
			Embolism incidence	4.88 ± 0.06	0.010
			Catheter disconnection rate	4.86 ± 0.08	0.015
			Incidence of catheterization	4.87 ± 0.08	0.016
			Embolism incidence	4.89 ± 0.05	0.012
			Catheter disconnection rate	4.86 ± 0.08	0.016
	Adverse event	4.82 ± 0.12	Incidence of catheterization	4.87 ± 0.08	0.016

### 3.5 Comparison of catheter infection rates between groups

The incidence of catheter infections was significantly lower in the observation group compared to the control group. Specifically, the observation group reported an infection rate of 3.12%, whereas the control group had a markedly higher rate of 16.12%. This difference was statistically significant (*P* < 0.05), as illustrated in Table 4. These findings underscore the effectiveness of the implemented infection prevention and control indicator system in reducing catheter-related infections among patients undergoing CRRT. The results indicate a substantial improvement in patient safety and highlight the importance of standardized protocols in clinical practice (Table 4).

**TABLE 4:** Comparison of the incidence of catheter infection between the two groups [cases (%)].

Group	Cases	Infected	Not infected
Control group	62	10 (16.12)	52 (83.87)
Observation group	64	2 (3.12)	62 (96.87)
χ^2^	–	–	4.675
*P*	–	–	0.032

## 4 Discussion

The study demonstrated a significant reduction in catheter infection rates among patients in the observation group, where the incidence was just 3.12%, compared to 16.12% in the control group. This statistically significant difference underscores the effectiveness of the comprehensive infection prevention and control indicator system implemented in the observation group. The findings highlight not only the potential for improved patient outcomes through structured protocols but also the importance of ongoing training and adherence to best practices in managing CRRT patients. Overall, these results indicate a promising avenue for enhancing infection control measures in critical care settings.

### 4.1 Clinical implications

The development of infection prevention indicators for CRRT is crucial for improving clinical practice and patient outcomes. Implementing standardized protocols can reduce CRBSIs in hemodialysis patients (11). A systematic review protocol has been proposed to identify quality indicators for CRRT care, which will support safe and efficient delivery of this therapy (12). A risk prediction model for CLABSIs in CRRT patients has been developed, enabling early assessment and screening of high-risk groups (13). Experts have emphasized the importance of standardizing CRRT management and identifying quality indicators to address the variability in practice and high risk of adverse events (14). These efforts collectively contribute to improving overall care quality in critical care settings, potentially reducing infection rates, shortening hospital stays, and positively impacting patient health and healthcare costs.

### 4.2 Comparison with existing literature

The findings of this study align with existing literature that emphasizes the critical role of infection prevention in CRRT patients. Numerous studies have reported elevated infection rates in this population, underscoring the need for effective management strategies. Zhang (15) developed predictive models for CRBSI in CRRT patients, demonstrating good judgment ability and clinical significance. Yang et al. (16) evaluated the efficacy of risk management in CRRT nursing, finding that it reduced adverse events, complications, and CRRT duration while improving patient psychological status, quality of life, and treatment compliance. These studies collectively emphasize the importance of infection prevention and risk management strategies in CRRT patients to improve outcomes and reduce complications. Our results demonstrate a significant reduction in catheter infection rates in the observation group, supporting previous research advocating for structured intervention systems. However, unlike some studies that highlight multifactorial influences on infection rates, our findings suggest that a targeted indicator system can directly impact outcomes. This disparity indicates that while various factors contribute to infection risks, a focused approach can yield measurable benefits in clinical practice.

### 4.3 Study limitations

Despite the significant findings, this study has several limitations that warrant consideration. First, potential biases in sample selection could affect the results; the study was conducted at a single institution, which may not fully represent the diversity of CRRT patients across different settings. Additionally, the methodology relies on the Delphi technique for expert consensus, which, while rigorous, may introduce subjective biases based on the selection of experts and their perspectives. Furthermore, the generalizability of our findings may be limited. The specific criteria for patient inclusion and exclusion might not capture the complexities of all CRRT patients, particularly those with varying comorbidities or different healthcare environments. As such, while our indicator system showed promise within this cohort, further research in diverse populations and settings is needed to validate its broader applicability and effectiveness in infection control.

### 4.4 Future research directions

Future research should explore several key areas to enhance the understanding and effectiveness of the developed infection prevention indicator system. Long-term studies are essential to assess the sustained impact of the system on catheter infection rates and overall patient outcomes beyond the initial implementation phase. Additionally, investigations into the system’s adaptability and effectiveness across various healthcare settings—such as community hospitals or specialized units—could provide valuable insights into its broader applicability. Further research could also delve into the cost-effectiveness of implementing the indicator system, considering both financial implications and resource allocation in critical care environments. Lastly, examining the integration of emerging technologies, such as telehealth monitoring and data analytics, could enhance the system’s responsiveness and effectiveness in real-time infection control.

## 5 Conclusion

The findings of this study underscore the critical importance of implementing a comprehensive infection prevention indicator system for patients undergoing CRRT. The significant reduction in catheter infection rates observed in the observation group highlights the potential for structured protocols to enhance patient safety and outcomes in critical care settings. These results not only advocate for improved nursing practices but also point to the necessity for ongoing research to refine and adapt infection control measures. Continuous development of protocols is essential to keep pace with evolving healthcare challenges and to ensure the highest standards of care for vulnerable patient populations.

## Data Availability

The original contributions presented in this study are included in this article/supplementary material, further inquiries can be directed to the corresponding author.
